# Tanshinone *ΙΙ*A-Incubated Mesenchymal Stem Cells Inhibit Lipopolysaccharide-Induced Inflammation of N9 Cells through TREM2 Signaling Pathway

**DOI:** 10.1155/2022/9977610

**Published:** 2022-03-04

**Authors:** Nanqu Huang, Juan Huang, Fei Feng, Zhisheng Ba, Yuanyuan Li, Yong Luo

**Affiliations:** ^1^National Drug Clinical Trial Institution, Third Affiliated Hospital of Zunyi Medical University (The First People's Hospital of Zunyi), Zunyi, Guizhou, China; ^2^Department of Pharmacology and Chemical Biology, Institute of Medical Sciences, Shanghai Jiao Tong University School of Medicine, Shanghai, China; ^3^Department of Neurology, Third Affiliated Hospital of Zunyi Medical University (The First People's Hospital of Zunyi), Zunyi, Guizhou, China

## Abstract

Our previous study found that incubating mesenchymal stem cells (MSC) with tanshinone IIA (TIIA) before transplantation could significantly increase the inhibitory effect of MSC on neuroinflammation. Here, we investigated the possible mechanism of this effect. N9 cells and MSC were inoculated at a ratio of 1 : 1 into a Transwell coculture system. MSC were inoculated into the upper chamber, and N9 cells were inoculated into the lower chamber. In this experiment, N9 cells were treated with 1 *μ*g/mL lipopolysaccharide (LPS) for 24 hours to induce inflammation, MSC were treated with 10 *μ*M TIIA for 48 hours to prepare TIIA-incubated MSC (TIIA-MSC), and TREM2 siRNA was used to silence the *TREM2* gene in MSC. The changes in IL-1*β*, IL-6, and TNF-*α* were evaluated by Western blotting. We found that LPS significantly increased the levels of IL-1*β*, IL-6, and TNF-*α*. While both MSC and TIIA-MSC downregulated the levels of (*P* = 0.092, *P* = 0.002), IL-6 (*P* = 0.014, *P* < 0.001), and TNF-*α* (*P* = 0.044, *P* = 0.003), TIIA-MSC downregulated IL-6 more significantly (*P* = 0.026). In addition, silencing TREM2 reduced the ability of TIIA-MSC to attenuate IL-6 (*P* = 0.005) and TNF-*α* (*P* = 0.033). These data suggest that the enhanced anti-inflammatory effect of TIIA-MSC on LPS-induced N9 cells may be mediated through the TREM2 signaling pathway.

## 1. Introduction

Alzheimer's disease (AD) is a common degenerative disease of the central nervous system characterized by progressive cognitive dysfunction and behavioral impairment. Active investigation of the pathogenesis and treatment of AD has extremely important value and significance. In recent years, a large number of studies have shown that mesenchymal stem cells (MSC) have great potential in the treatment of AD. There are many possible mechanisms how MSC function in the treatment of AD, but they are largely related to the regulation of neuroinflammation by MSC: when inflammation is weak, MSC promote the immune response; when inflammation is too strong, MSC inhibit the immune response [1]. In addition, MSC can migrate toward injured tissue [2]. Based on these effects, MSC can be a promising treatment modality because of their anti-AD neuroinflammatory activity. Our previous research found that MSC are effective in improving the learning and memory ability of AD model rats by blocking neuronal apoptosis and reducing the neuroinflammation-related cytokines interleukin 1*β* (IL-1*β*), IL-6, and tumor necrosis factor (TNF-*α*) [3]. However, the application of MSC still faces problems and challenges. MSC are prone to aging after *in vitro* expansion, and the environmental factors may suppress their ability to differentiate into specific mature cells and regulate inflammation [4].

To solve these problems, our research group originally used the biologically active ingredient tanshinone IIA (TIIA) isolated from the traditional Chinese medicine *Salvia miltiorrhiza* Bunge (Danshen) before MSC transplantation. Danshen is a well-known medicinal herb with a long history of clinical application for treating cardiovascular diseases, cancer, and osteoporosis [5, 6]. TIIA is the main active component of Danshen and is known to significantly improve blood circulation and delay tumor progression [7]. Our previous research found that pretreatment with TIIA can improve the damage to hippocampal neurons in rats caused by oxygen-glucose deprivation (OGD) [8]. OGD mimics the environmental changes MSC are exposed to during expansion and transplantation. Therefore, we decided to test TIIA to solve this problem.

We found that TIIA enhanced the inhibition of neuroinflammation by MSC and that TIIA-treated MSC (TIIA-MSC) exerted better neuroprotection [3]. However, the specific mechanism is not clear. Interestingly, studies have shown that TREM2-modified MSC have better neuroprotective effects [9]. TREM2 is a type of immunoglobulin-like receptor that is highly expressed on microglia. After binding to ligands, its signal is mediated by dead cell activation receptor-related protein (DAP12), which plays a negative regulatory role in autoimmunity and inflammation [10]. TREM2 has two physiological functions, inhibiting inflammation and promoting the phagocytosis of apoptotic neurons [11]. However, this effect is complicated. Studies have shown the deleterious role of TREM2 deficiency in the development of AD [12]. However, there are also opinions that with the progression of amyloid pathology, the upregulation of TREM2 reduces inflammation, promotes immune tolerance, and promotes microglia to clear A*β* [13]. Upregulation of TREM2 may help prevent AD, and downregulation of TREM2 without affecting the physiological function of TREM2 may be a strategy for the treatment of AD [13]. Therefore, we speculated that the enhanced activity of TIIA-MSC against neuroinflammation may also be through the regulation of TREM2. In this study, we used a Transwell coculture system and TREM2 siRNA to further explore the possible mechanisms of the elevated antineuroinflammation activity of TIIA-MSC.

## 2. Materials and Methods

### 2.1. Materials

N9 cells, which are a microglial cell line derived from the mouse brain and have many phenotypic characteristics similar to those of primary mouse microglial cells [14], were purchased from Beijing Zhongke Quality Inspection Biotechnology (ZKCC-X1877). TIIA (purity > 99.37%) was obtained from MedChemExpress. Following reagents and instruments were obtained from indicated sources: LPS (L4391, Sigma), RPMI 1640 (61870036, Gibco), medium for mesenchymal stem cells (mubmx-03011-440, Cyagen), DMEM (10566016, Gibco), trypsin-EDTA (T1300, Solarbio), PBS (KGB5001, Keygenbio), FBS (E600001-0500, BBI Life Sciences), Penicillin–Streptomycin Liquid (P1400, Solarbio), OPTI-MEM® (31985-062, Gibco), Lipofectamine™3000 (L3000015, Invitrogen™), TREM2 RNA interference vector and negative control siRNA vector (Generalbiol), CD45 PerCP (103129, Biolegend), CD29 PE (102207, Biolegend), CD90 PE-CY7 (105325, Biolegend), NovoCyte™ Flow Cytometer (NovoCyte 2060R, ACEA BIO Co., LTD), automatic microplate reader (WD-2102B, Beijing Liuyi Biotechnology Co., Ltd.), TRIzol Reagent (CW0580S, Cwbiotech), HiFiScript 1st Strand cDNA Synthesis Kit (CW2569M, Cwbiotech), UltraSYBR Mixture (CW0957M, Cwbiotech), Real-Time Fluorescence PCR (CFX Connect™, Bio-Rad Laboratories), RIPA (C1053, Applygen Technologies), BCA Protein Assay Kit (CW0014S, Cwbiotech), Marker (#26617, Thermo Scientific), PVDF (IPVH00010, Merck), skimmed milk powder (P1622, Applygen Technologies), Mouse Monoclonal Anti-GAPDH (1/2000, TA-08, Zsbio), Rabbit Anti TREM2 (1/1000, DF12529, Affinity), Rabbit Anti IL-1*β* (1/1000, bs-0812R, Bioss), Rabbit Anti TNF-*α* (1/500, AF7014, Affinity), goat anti-mouse immunoglobulin (Ig)G H&L (1/2000, ZB-2305, Zsbio), and goat anti-rabbit immunoglobulin (Ig)G H&L (1/2000, ZB-2301, Zsbio).

### 2.2. Preparation and Identification of MSC

C57BL/6 mice were purchased from ZHBY Biotech, Jiangxi, China (grade: specific pathogen-free, SCXK 2019-0004) and housed at 22-23°C with a 12-hour light/dark cycle. The mice were sacrificed by cervical dislocation and soaked in 75% ethanol for 5 minutes. The tibia and humerus of mice were removed under aseptic conditions. The periosteum and muscle tissue were dissected, and the tibia and humerus were washed repeatedly with DPBS containing 1% penicillin-streptomycin. Next, the medullary cavity was flushed thoroughly with DMEM complete medium to harvest bone marrow cells. Collected cells were centrifuged, the supernatant was discarded after centrifugation (1200 rpm, 5 min, 4°C), and the pellet was resuspended in DMEM complete medium. The bone marrow cells were spread evenly on plates and cultured in a humidified atmosphere with 5% CO_2_ at 37°C [15]. All procedures were performed with the approval of the Animal Experimental Ethical Committee of Zunyi Medical University (no. (2019)2-231, 11 Mar 2019).

MSC were identified by flow cytometry using antibodies against CD29, CD45, and CD90. Briefly, the MSC were washed twice with ice-cold PBS. Subsequently, 1 − 3 × 10^6^ cells were mixed with the antibodies CD45 PerCP, CD29 PE, and CD90 PE-CY7. The cell suspensions were incubated at room temperature for 20 min in the dark. After centrifugation of the mixture, the cells were washed three times with PBS, resuspended, and analyzed using flow cytometry.

### 2.3. Cell Culture and Processing

**Figure 1:**
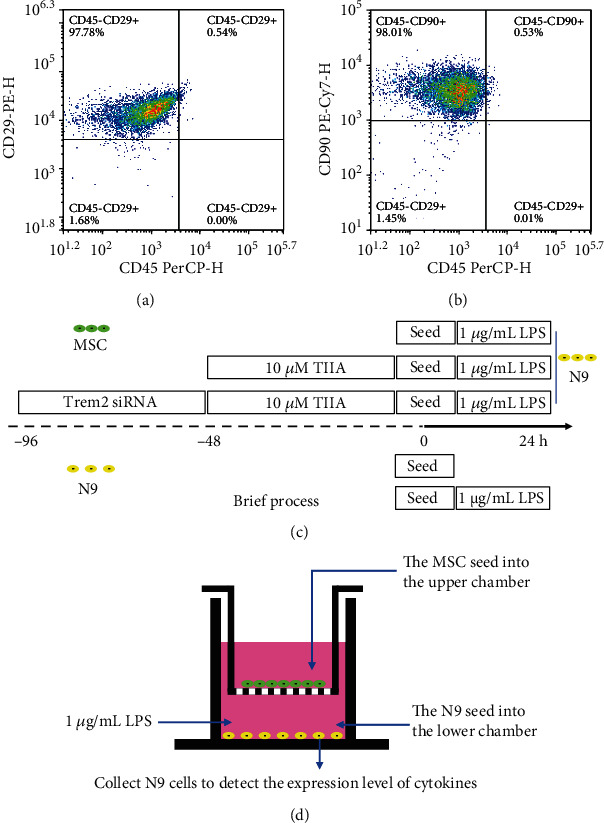
The identification of MSC by flow cytometry and the schematic representation of the study design. (a, b) Flow cytometry was used to detect the surface antigens of MSC: CD29 (97.78%), CD90 (98.01%), and CD45 (0.54%). (c) Brief descriptions of the experimental procedures and experimental conditions. (d) Transwell system used in the present study.

N9 cells were cultured in DMEM complete medium containing 10% FBS and 1% penicillin-streptomycin. The cells were subcultured when they reached 80-90% confluence. N9 cells and MSC were cultured in an indirect coculture system using Transwell filters at a cell ratio of 1 : 1. In this study, MSC were inoculated into the upper chamber, and N9 cells were inoculated into the lower chamber (Figure 1(d)). N9 cells were treated with 1 *μ*g/mL LPS for 24 hours to induce inflammation. MSC were treated with 10 *μ*M TIIA for 48 hours to prepare TIIA-MSC. In this study, we used MSC modified with TIIA, not a mixture of TIIA and MSC. Therefore, TIIA was discarded after TIIA treatment. The TREM2 gene of MSC was silenced by TREM2 siRNA by treating the cells with siRNA for 48 hours. The siRNA was discarded after the treatment. The details of the experimental conditions and specific procedures used in this study are shown in Figure 1(c).

### 2.4. Cell Transfection

When the MSC reached 80-90% confluence, the cell culture medium was replaced with 1 mL of serum-free medium. To prepare transfection reagents, 125 *μ*L Opti-MEM was added to 2 sterilized EP tubes. Five microliter Lipofectamine 3000 was added to one of the tubes, 12.5 *μ*L siRNA (the dry siRNA powder was dissolved in DEPC water; 125 *μ*L/1 OD) was added to another, and the tubes were mixed and incubated at room temperature for 5 min. Next, the content of the above two EP tubes was combined and incubated at room temperature for 15 min. Afterward, the mixture was applied to the MSC dropwise. Six hours after transfection, 1 mL of complete medium with a serum content of 20% was added to the six-well plate. Finally, the transfection efficiency was determined by RT-qPCR and Western blotting at 48 hours after transfection [16].

### 2.5. RT-qPCR

Forty-eight hours after transfection, the total RNA from cultured cell samples was extracted using TRIzol lysis buffer through a series of rinse, elution, and centrifugation steps. The concentration and purity of RNA (OD260/OD280) were measured with a UV-Vis spectrophotometer. cDNA was synthesized by reverse transcription using a HiFiScript cDNA first-strand synthesis kit. A fluorescent PCR machine was used to perform fluorescent quantitative PCR. The composition of the reaction mixture was as follows: RNase Free dH_2_O 9.5 *μ*L, cDNA 1 *μ*L, upstream primer 1 *μ*L, downstream primer 1 *μ*L, and 2 × SYBR Green PCR Master Mix 12.5 *μ*L. The reaction steps were as follows: predenaturation at 95°C 10 min; followed by 40 cycles of denaturation at 95°C for 10 s; annealing at 58°C for 30 s; extension at 72°C for 30 s. The primer sequences were as follows: TREM2 forward primer 5′-CATGTACTTATGACGCCTTGAA-3′, reverse primer 5′-TCTGCGATGACT GTGCTCC-3′; GAPDH forward primer: 5′-TCAACGGCACAGTCAAGG-3′, reverse primer 5′-TGAGCCCTTCCACGATG-3′. GAPDH was used as an internal control, and the relative expression of TREM2 was calculated according to the 2^-△△Ct^ method.

### 2.6. Western Blotting

N9 cells were incubated with lysis buffer for 30 min at 4°C, and insoluble material was removed by centrifugation at 12,000 rpm for 10 min at 4°C. The supernatant was carefully collected to obtain the total protein. The protein concentration was determined using the BCA kit. Equal amounts of protein were separated by sodium dodecylbenzene sulfonate gel electrophoresis (SDS-PAGE) for 2 hours and transferred to PVDF membranes with a constant current of 300 mA for 80 min. The blots were blocked with fat-free milk solution in TBST for 1 hour at room temperature. The blots were washed with TBST three times and incubated with the primary antibody overnight at 4°C. The blots were washed with TBST three times for 5 min each time followed by incubation with the corresponding secondary antibody solution for 2 hours at room temperature. Then, PVDF membranes were washed with TBST three times and visualized using ECL solution. The gray value of each antibody band was analyzed with Quantity One software version 4.6.1 software.

### 2.7. Statistical Analyses

All data were statistically analyzed with SPSS 22.0 and expressed as the mean ± SEM from at least three independent experiments. Significant differences between groups were analyzed by one-way ANOVA. *P* < 0.05 was considered significant.

## 3. Results

### 3.1. Identification of MSC

To identify whether the isolated and cultured cells were MSC, we used flow cytometry to detect MSC markers. The flow cytometry results showed that the cells expressed CD29 (97.78%) and CD90 (98.01%) and almost no expression of the hematopoietic cell marker CD45 (0.54%) (Figure 1). These results are consistent with the standard surface markers of mouse MSC.

### 3.2. Transfection Efficiency Test

**Figure 2:**
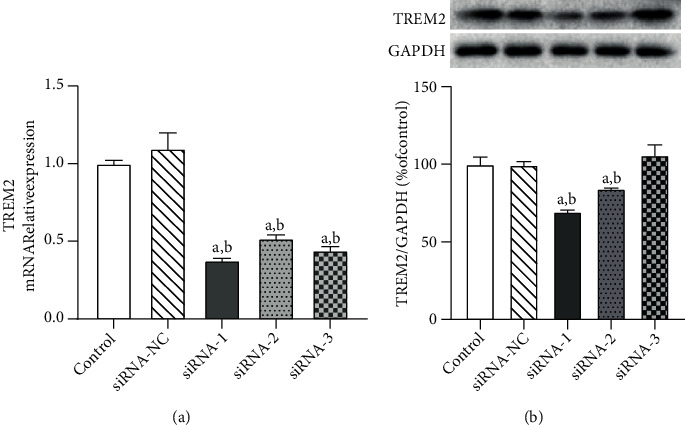
RT-qPCR and Western blotting analyses of TREM2 siRNA transfection efficiency. (a) Relative expression levels of TREM2 mRNA. (b) Expression levels of TREM2 protein. ^a^*P* < 0.05 vs. control, ^b^*P* < 0.05 vs. siRNA-NC, mean ± SEM, *n* = 3.

In this study, three TREM2 interference sequences (siRNA-1, siRNA-2, and siRNA-3) were designed and transfected into MSC. After 48 h, the transfection efficiency was measured by RT-qPCR and Western blotting (Figure 2). Compared with siRNA-NC, TREM2 interference (siRNA-1, siRNA-2, and siRNA-3) significantly reduced the mRNA expression level of TREM2 (Figure 2(a)); in addition, Western blotting results showed that, compared with siRNA-NC, siRNA-1 and siRNA-2 downregulated the levels of TREM2 protein, and the effect of siRNA-1 was more pronounced than that of siRNA-2 (Figure 2(b)). The results showed that transfection of TREM2 siRNA was successful, and because siRNA-1 showed the best downregulation, it was used for subsequent experiments.

### 3.3. Changes in the Levels of Inflammatory Factors

**Figure 3:**
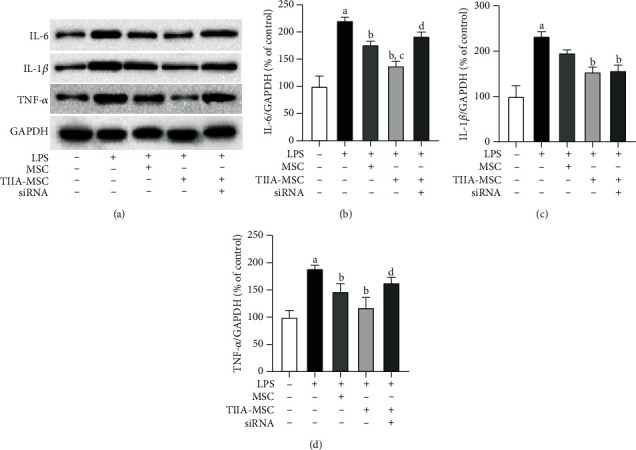
Inflammatory factor (IL-1*β*, IL-6, and TNF-*α*) protein expression. (a) Representative Western blotting results for IL-1*β*, IL-6, TNF-*α*, and GAPDH. (b) Relative protein expression of IL-6. (c) Relative protein expression of IL-1*β*. (d) Relative protein expression of TNF-*α*. ^a^*P* < 0.05 vs. control, ^b^*P* < 0.05 vs. LPS, ^c^*P* < 0.05 vs. LPS + MSC, ^d^*P* < 0.05 vs. LPS + TIIA − MSC, mean ± SEM, *n* = 3.

Western blotting was used to determine the expression levels of the inflammatory factors IL-1*β*, IL-6, and TNF-*α*, and the results are shown in Figure 3. Compared with the control group, LPS significantly increased the protein levels of IL-1*β*, IL-6, and TNF-*α*, indicating that LPS stimulated the inflammatory response of N9 cells. Compared with the LPS-treated cells, both MSC and TIIA-MSC downregulated the expression levels of IL-1*β*, IL-6, and TNF-*α*, but TIIA-MSC showed a more pronounced downregulatory effect on inflammatory factors. Compared with MSC, TIIA-MSC significantly reduced IL-6. After the TREM2 gene was silenced using TREM2 siRNA in MSC, the ability of TIIA-MSC to reduce inflammatory factors in N9 cells was suppressed. Compared with TIIA-MSC, TREM2 siRNA significantly suppressed the reduction of IL-6 and TNF-*α* levels, but this effect seemed to be less obvious in the regulation of IL-1*β* expression.

## 4. Discussion

TREM2 is a type of immunoglobulin-like receptor that is highly expressed on microglia. Large-scale gene sequencing in the population has shown that a genetic mutation of TREM2 is closely associated with AD [17]. In AD, TREM2 can initiate microglial responses by maintaining cell energy and biosynthesis [18], which participates in the response of microglia to A*β* plaque deposition [19]. Interestingly, a large amount of its extracellular soluble fragment (sTREM2) is present in the cerebrospinal fluid of AD patients and is closely related to the tau protein level, which is one of the characteristic features in the pathology of AD. sTREM2 can increase the survival rate of microglia in the brain by regulating phosphatidylinositol 3-kinase/protein kinase B (PI3K/Akt), which can induce inflammation and enhance the viability of microglia [20]. *In vitro*, upregulation of TREM2 expression reduced the number of apoptotic neurons and increased the expression of some anti-inflammatory factors. Conversely, downregulation of TREM2 expression increased the number of apoptotic neurons and promoted the expression of proinflammatory factors [21]. *In vivo*, intracerebroventricular injection of TREM2 overexpression lentivirus can promote the transformation of microglia to the M2 type and reduce neuronal apoptosis [22].

Interestingly, TREM2 is also expressed in MSC, and the activation of TREM2 can inhibit the immune activation of MSC and promote their differentiation [23]. .TREM2-modified MSC have better neuroprotective effects [9]. This study found that compared with MSC, TIIA-MSC significantly reduced the expression levels of IL-6, and the downregulatory effect was more significant. At the same time, silencing the *TREM2* gene in MSC greatly suppressed the ability of TIIA-MSC to reduce inflammatory factors in N9 cells. Compared with TIIA-MSC, TREM2 siRNA significantly attenuated the decline in IL-6 and TNF-*α*, but this effect seemed to be less obvious for IL-1*β*. We suspect that the effect of TIIA-MSC on IL-1*β* was not apparent because the affected part of IL-1*β* is secreted into the medium, and our assay did not detect this part of IL-1*β*. Through the present data, it can be preliminarily surmised that the TREM2 signaling pathway is the key to the neuroinflammatory regulation of TIIA-MSC.

MSC can mediate and regulate the balance of the immune response through paracrine mechanisms and the interaction between MSC and immune cells. Bone marrow-derived MSC exosomes play an immunomodulatory role in various autoimmune-related diseases [24] and can reduce tissue damage and promote tissue repair [25]. Therefore, we speculate that the enhanced inhibitory activity of TIIA-MSC may be related to the regulation of the TREM2 signaling pathway to affect the secretion of exosomes. In addition, the role of MSC in regulating neuroinflammation may be due to their directed differentiation into microglia with specific phenotypes [26]. Therefore, another possible reason for the function of TIIA-MSC is that they promote the expression of TREM2 in MSC and further differentiate into microglia with high TREM2 expression, thereby negatively regulating neuroinflammation and exerting a protective effect. However, this study also has certain limitations. For example, we did not test microglial proliferation, anti-inflammatory cytokines, or chemokines, such as CCL2, CXCL1, and IL-10. These will be assessed in our future studies.

## 5. Conclusions

The superior efficiency of TIIA-MSC in inhibiting the inflammatory response of N9 cells induced by LPS is related to the regulation of the TREM2 signaling pathway. This not only provides support for the application of TIIA to pretreat MSC but also provides new ideas for applying MSC for the treatment of neuroinflammation-related AD.

## Data Availability

The authors declare that all the data supporting the findings in this study are available from the corresponding author through email on reasonable request.
